# A Study of the Radiation Tolerance of CVD Diamond to 70 MeV Protons, Fast Neutrons and 200 MeV Pions

**DOI:** 10.3390/s20226648

**Published:** 2020-11-20

**Authors:** Lukas Bäni, Andreas Alexopoulos, Marina Artuso, Felix Bachmair, Marcin Ryszard Bartosik, Helge Christoph Beck, Vincenzo Bellini, Vladimir Belyaev, Benjamin Bentele, Alexandre Bes, Jean-Marie Brom, Gabriele Chiodini, Dominik Chren, Vladimir Cindro, Gilles Claus, Johann Collot, John Cumalat, Sébastien Curtoni, Anne Evelyn Dabrowski, Raffaello D’Alessandro, Denis Dauvergne, Wim De Boer, Christian Dorfer, Marc Dünser, Gerald Eigen, Vladimir Eremin, Jacopo Forneris, Laurent Gallin-Martel, Marie-Laure Gallin-Martel, Kock Kiam Gan, Martin Gastal, Abderrahman Ghimouz, Mathieu Goffe, Joel Goldstein, Alexander Golubev, Andrej Gorišek, Eugene Grigoriev, Jörn Grosse-Knetter, Aidan Grummer, Bojan Hiti, Dmitry Hits, Martin Hoeferkamp, Jérôme Hosselet, Fabian Hügging, Chris Hutson, Jens Janssen, Harris Kagan, Keida Kanxheri, Richard Kass, Mladen Kis, Gregor Kramberger, Sergey Kuleshov, Ana Lacoste, Stefano Lagomarsino, Alessandro Lo Giudice, Ivan López Paz, Eric Lukosi, Chaker Maazouzi, Igor Mandić, Sara Marcatili, Alysia Marino, Cédric Mathieu, Mauro Menichelli, Marko Mikuž, Arianna Morozzi, Francesco Moscatelli, Joshua Moss, Raymond Mountain, Alexander Oh, Paolo Olivero, Daniele Passeri, Heinz Pernegger, Roberto Perrino, Federico Picollo, Michal Pomorski, Renato Potenza, Arnulf Quadt, Fatah Rarbi, Alessandro Re, Michael Reichmann, Shaun Roe, Olivier Rossetto, Diego Alejandro Sanz Becerra, Christian J. Schmidt, Stephen Schnetzer, Silvio Sciortino, Andrea Scorzoni, Sally Seidel, Leonello Servoli, Dale Shane Smith, Bruno Sopko, Vit Sopko, Stefania Spagnolo, Stefan Spanier, Kevin Stenson, Robert Stone, Bjarne Stugu, Concetta Sutera, Michael Traeger, William Trischuk, Marco Truccato, Cristina Tuvè, Jaap Velthuis, Stephen Wagner, Rainer Wallny, Jianchun Wang, Norbert Wermes, Jayashani Wickramasinghe, Mahfoud Yamouni, Justas Zalieckas, Marko Zavrtanik, Kazuhiko Hara, Yoichi Ikegami, Osamu Jinnouchi, Takashi Kohriki, Shingo Mitsui, Ryo Nagai, Susumu Terada, Yoshinobu Unno

**Affiliations:** 1 Department of Physics, ETH Zürich, 8093 Zürich, Switzerland; felix.bachmair@gmail.com (F.B.); dorfer@phys.ethz.ch (C.D.); dmitry.hits@phys.ethz.ch (D.H.); micha.reichmann@gmail.com (M.R.); sandiego@phys.ethz.ch (D.A.S.B.); rainer.wallny@phys.ethz.ch (R.W.); 2 CERN, 1211 Geneva, Switzerland; a.alexopoulos@cern.ch (A.A.); marcin.bartosik@cern.ch (M.R.B.); anne.evelyn.dabrowski@cern.ch (A.E.D.); marc.dunser@cern.ch (M.D.); martin.gastal@cern.ch (M.G.); heinz.pernegger@cern.ch (H.P.); shaun.roe@cern.ch (S.R.); 3 Department of Physics, Syracuse University, Syracuse, NY 13210, USA; artuso@physics.syr.edu (M.A.); raym@physics.syr.edu (R.M.); jwang@physics.syr.edu (J.W.); 4 Institute of Physics, Universität Göttingen, D-37077 Göttingen, Germany; helge-christoph.beck@phys.uni-goettingen.de (H.C.B.); jgrosse1@uni-goettingen.de (J.G-K.); aquadt@uni-goettingen.de (A.Q.); 5 Department of Physics and Astronomy, INFN/University of Catania, 95123 Catania, Italy; vincenzo.bellini@ct.infn.it (V.B.); renato.potenza@libero.it (R.P.); concetta.sutera@ct.infn.it (C.S.); cristina.tuve@ct.infn.it (C.T.); 6 MEPHI Institute, 115409 Moscow, Russia; belyaev@mephi.ru; 7 Physics Department, University of Colorado, Boulder, CO 80309, USA; Benjamin.Bentele@colorado.edu (B.B.); jcumalat@pizero.colorado.edu (J.C.); amarino@colorado.edu (A.M.); Kevin.Stenson@colorado.edu (K.S.); Stephen.Wagner@colorado.edu (S.W.); 8 LPSC-Grenoble, 38026 Grenoble, France; bes@lpsc.in2p3.fr (A.B.); collot@in2p3.fr (J.C.); curtoni@lpsc.in2p3.fr (S.C.); denis.dauvergne@lpsc.in2p3.fr (D.D.); laurent.gallin-martel@lpsc.in2p3.fr (L.G.-M.); mlgallin@lpsc.in2p3.fr (M.-L.G.-M.); ghimouz@lpsc.in2p3.fr (A.G.); ana.lacoste@ujf-grenoble.fr (A.L.); sara.marcatili@lpsc.in2p3.fr (S.M.); rarbi@lpsc.in2p3.fr (F.R.); rossetto@lpsc.in2p3.fr (O.R.); mahfoud.yamouni@lpsc.in2p3.fr (M.Y.); 9 IPHC, F-67000 Strasbourg, France; brom@in2p3.fr (J.-M.B.); gilles.claus@iphc.cnrs.fr (G.C.); mathieu.goffe@iphc.cnrs.fr (M.G.); jerome.hosselet@iphc.cnrs.fr (J.H.); chaker.maazouzi@iphc.cnrs.fr (C.M.); cedric.mathieu@iphc.cnrs.fr (C.M.); 10 INFN-Lecce, 73100 Lecce, Italy; gabriele.chiodini@le.infn.it (G.C.); roberto.perrino@le.infn.it (R.P.); stefania.spagnolo@le.infn.it (S.S.); 11 Department of Physics, Czech Technical University, 166 29 Prague, Czech Republic; dominik.chren@fs.cvut.cz (D.C.); bruno.sopko@cern.ch (B.S.); vit.sopko@cern.ch (V.S.); 12 Department of Physics, Jožef Stefan Institute, University of Ljubljana, SI-1000 Ljubljana, Slovenia; vladimir.cindro@ijs.si (V.C.); andrej.gorisek@cern.ch (A.G.); bojan.hiti@ijs.si (B.H.); gregor.kramberger@ijs.si (G.K.); igor.mandic@ijs.si (I.M.); marko.mikuz@cern.ch (M.M.); marko.zavrtanik@ijs.si (M.Z.); 13 Department of Physics and Astronomy, INFN/University of Florence, 50145 Florence, Italy; candi@fi.infn.it (R.D.); lagomarsino@fi.infn.it (S.L.); sciortino@fi.infn.it (S.S.); 14 Institut für Experimentelle Kernphysik, Universität Karlsruhe, D-76049 Karlsruhe, Germany; wim.de.boer@kit.edu; 15 Department of Physics and Technology, University of Bergen, 5007 Bergen, Norway; gerald.eigen@uib.no (G.E.); bjarne.stugu@uib.no (B.S.); justas.zalieckas@cern.ch (J.Z.); 16 Ioffe Institute, 194021 St. Petersburg, Russia; vladimir.eremin@cern.ch; 17 Dipartimento di Fisica, University of Torino, 10125 Torino, Italy; forneris@to.infn.it (J.F.); alessandro.logiudice@unito.it (A.L.G.); paolo.olivero@unito.it (P.O.); federico.picollo@unito.it (F.P.); alessandro.re@unito.it (A.R.); marco.truccato@unito.it (M.T.); 18 Department of Physics, The Ohio State University, Columbus, OH 43210, USA; gan.1@osu.edu (K.K.G.); kagan.1@osu.edu (H.K.); kass.1@osu.edu (R.K.); ssmith@mps.ohio-state.edu (D.S.S.); 19 School of Physics, University of Bristol, Bristol BS8 1TL, UK; joel.goldstein@bristol.ac.uk (J.G.); chris.hutson@bristol.ac.uk (C.H.); jaap.velthuis@bristol.ac.uk (J.V.); 20 ITEP, 117218 Moscow, Russia; alexander.golubev@itep.ru (A.G.); eugene.grigoriev@cern.ch (E.G.); serguei.koulechov@cern.ch (S.K.); 21 Department of Physics and Astronomy, University of New Mexico, Albuquerque, NM 87131, USA; agrummer@unm.edu (A.G.); martin@phys.unm.edu (M.H.); seidel@unm.edu (S.S.); jwickramasinghe@unm.edu (J.W.); 22 Physikalisches Institut, Universität Bonn, 53115 Bonn, Germany; huegging@physik.uni-bonn.de (F.H.); jens.janssen@cern.ch (J.J.); wermes@uni-bonn.de (N.W.); 23 INFN-Perugia, 06123 Perugia, Italy; keida.kanxheri@pg.infn.it (K.K.); mauro.menichelli@pg.infn.it (M.M.); arianna.morozzi@gmail.com (A.M.); moscatelli@iom.cnr.it (F.M.); daniele.passeri@unipg.it (D.P.); andrea.scorzoni@unipg.it (A.S.); leonello.servoli@pg.infn.it (L.S.); 24 GSI Helmholtzzentrum für Schwerionenforschung, 64291 Darmstadt, Germany; m.kis@gsi.de (M.K.); c.j.schmidt@gsi.de (C.J.S.); m.traeger@gsi.de (M.T.); 25 Manchester School of Physics and Astronomy, University of Manchester, Manchester M13 9PL, UK; ivan.lopez.paz@cern.ch (I.L.P.); alexander.oh@cern.ch (A.O.); 26 Department of Nuclear Engineering, University of Tennessee, Knoxville, TN 37996, USA; elukosi@utk.edu (E.L.); sspanier@utk.edu (S.S.); 27 Physics Faculty, California State University, Sacramento, CA 95819, USA; joshua.moss@cern.ch; 28 CEA-LIST Technologies Avancées, F91191 Gif-sur-Yvette, France; michal.pomorski@cea.fr; 29 Department of Physics and Astronomy, Rutgers University, Piscataway, NJ 08854, USA; steves@physics.rutgers.edu (S.S.); stone@physics.rutgers.edu (R.S.); 30 Department of Physics, University of Toronto, Toronto, ON M5S 1A7, Canada; william@physics.utoronto.ca; 31 Faculty of Pure and Applied Sciences, University of Tsukuba, Tsukuba 305-8571, Japan; hara@physics.px.tsukuba.ac.jp (K.H.); smitsui@staff.kanazawa-u.ac.jp (S.M.); 32 KEK, High Energy Accelerator Research Organization, Tsukuba, Ibaraki 305-0801, Japan; ikegami@post.kek.jp (Y.I.); takashi.kohriki@kek.jp (T.K.); susumu.terada@kek.jp (S.T.); yoshinobu.unno@kek.jp (Y.U.); 33 School of Science, Tokyo Institute of Technology, Tokyo 152-8551, Japan; jinnouchi@phys.titech.ac.jp (O.J.); rnagai@hepburn.s.chiba-u.ac.jp (R.N.)

**Keywords:** Chemical Vapor Deposition, single-crystalline diamond, polycrystalline diamond, charge collection distance, mean drift path, schubweg, radiation tolerance, radiation damage constant

## Abstract

We measured the radiation tolerance of commercially available diamonds grown by the Chemical Vapor Deposition process by measuring the charge created by a 120 GeV hadron beam in a 50 μm pitch strip detector fabricated on each diamond sample before and after irradiation. We irradiated one group of samples with 70 MeV protons, a second group of samples with fast reactor neutrons (defined as energy greater than 0.1 MeV), and a third group of samples with 200 MeV pions, in steps, to (8.8±0.9) × 10^15^ protons/cm^2^, (1.43±0.14) × 10^16^ neutrons/cm^2^, and (6.5±1.4) × 10^14^ pions/cm^2^, respectively. By observing the charge induced due to the separation of electron–hole pairs created by the passage of the hadron beam through each sample, on an event-by-event basis, as a function of irradiation fluence, we conclude all datasets can be described by a first-order damage equation and independently calculate the damage constant for 70 MeV protons, fast reactor neutrons, and 200 MeV pions. We find the damage constant for diamond irradiated with 70 MeV protons to be 1.62±0.07(stat)±0.16(syst)× 10^−18^ cm^2^/(p μm), the damage constant for diamond irradiated with fast reactor neutrons to be 2.65±0.13(stat)±0.18(syst)× 10^−18^ cm^2^/(n μm), and the damage constant for diamond irradiated with 200 MeV pions to be 2.0±0.2(stat)±0.5(syst)× 10^−18^ cm^2^/(π μm). The damage constants from this measurement were analyzed together with our previously published 24 GeV proton irradiation and 800 MeV proton irradiation damage constant data to derive the first comprehensive set of relative damage constants for Chemical Vapor Deposition diamond. We find 70 MeV protons are 2.60 ± 0.29 times more damaging than 24 GeV protons, fast reactor neutrons are 4.3 ± 0.4 times more damaging than 24 GeV protons, and 200 MeV pions are 3.2 ± 0.8 more damaging than 24 GeV protons. We also observe the measured data can be described by a universal damage curve for all proton, neutron, and pion irradiations we performed of Chemical Vapor Deposition diamond. Finally, we confirm the spatial uniformity of the collected charge increases with fluence for polycrystalline Chemical Vapor Deposition diamond, and this effect can also be described by a universal curve.

## 1. Introduction

Diamond-based radiation monitors are now routinely used in high-energy physics experiments (e.g., at the Large Hadron Collider (LHC) [1]). Their role has become critical in protecting more sensitive devices against extreme beam conditions and in contributing to a precision measurement of the luminosity the accelerator delivers. As a result, quantifying the radiation resistance, or damage constant, of diamond is critical to its use in future upgraded high energy facilities [2,3].

In a previously published paper [4], we described the methodology we used to measure the damage constants of polycrystalline CVD (pCVD) diamond and single-crystalline CVD (scCVD) diamond irradiated with 800 MeV and 24 GeV protons. The work described herein used the same methodology to measure the damage constants of Chemical Vapor Deposition (CVD) diamond irradiated with 70 MeV protons, fast reactor neutrons with energies greater than 0.1 MeV, and 200 MeV pions. In addition, in this manuscript, we derive universal curves for the damage as a function of fluence and the full width at half maximum divided by its most probable value (FWHM/MP) of the signal spectrum as a function of fluence which may then be used to predict the effects of radiation on any planned diamond detectors.

## 2. Sample Preparation

Two types of CVD diamond were used in this work. The first is single-crystalline, which, as the name implies, is ideally one single diamond crystal devoid of grains and grain boundaries. High purity single-crystalline material has been shown to collect the full charge deposited in the material but the material area is currently limited to ∼7 mm × 7 mm. The second is poly-crystalline, which, as the name implies, is made up of a collection of randomly oriented individual crystal grains and thus grain boundaries. In poly-crystalline material, the collected charge is less than the deposited charge due to the grain boundaries and their associated dislocations and traps. A high quality, 500 μm thick, pCVD diamond collects approximately half of the deposited charge but can be grown in very large areas up to 15 cm diameter wafers. To quantify the radiation tolerance of scCVD diamond and pCVD diamond, we used a series of commercially available diamonds for this study [5].

In preparing the diamond devices for testing, a 50 μm pitch strip detector was fabricated on each sample. The same strip width and strip detector pitch was used for both pCVD and scCVD diamond. Before metalization, each sample was cleaned with a multi-step hot acid cleaning followed by an oxygen plasma etch to clean the samples and terminate the surface with oxygen [6]. Both sides of the diamond were metalized with 500 Å Cr and 2000 Å Au. A single pad was fabricated on the bias side using photolithographic techniques. Then, 25 μm wide strips with a 25 μm gap between strips were fabricated with photolithographic techniques on the readout side producing a device with 50 μm pitch. A guard ring enclosed the strip pattern at the same potential as the strips to minimize any edge or surface currents from being picked up by the individual electronic channels. After metalization, each device was annealed at 400 ${}^{{}^{\circ}}C$ for 4 min in an $N_{2}$ atmosphere. The bias electrode side of the detector was attached with silver paint [7] to a ceramic hybrid containing a bias pad and RC bias filter circuit to power the device. A G-10 printed circuit board was used to house a 128-channel IDE AS VA2.2 readout chip [8] and was mounted next to the ceramic hybrid. Each diamond strip detector channel was directly wire bonded to a VA2.2 input pad. The signal return path and the bias voltage return path were connected together on the G-10 board near the VA2.2 integrated circuit to minimize the noise. Each of the 128 VA2.2 amplifier channels includes a charge sensitive preamplifier, followed by a CR-RC signal shaper. The signal rise time was set to 2 μs. In the configuration described above, a total noise per channel of typically ∼100 *e* was observed [4].

## 3. Sample Description

To determine the radiation tolerance of CVD diamond against protons, neutrons, and pions, seven samples with different properties were measured before and after irradiation. Two samples were irradiated with 70 MeV protons in steps up to a fluence of 8.8 × 10^15^ *p*/cm^2^, two samples were exposed to fast reactor neutrons up to a fluence of 14.3 × 10^15^ *n*/cm^2^, and three samples were irradiated with 200 MeV pions up to a fluence of 0.65 × 10^15^ π/cm^2^. After each irradiation, a 50 μm pitch strip detector was fabricated on each sample and each device was characterized in a 120 GeV hadron beam. The properties of the 70 MeV proton irradiated samples are listed in Table 1, Table 2 shows the properties of the fast neutron irradiated samples, and Table 3 presents the properties of the pion irradiated samples. The initial unirradiated signal response of each sample was determined before any irradiations by fabricating a single pad detector on each diamond sample and measured by using a calibrated setup [9] with a ^90^Sr β-source.

## 4. Sample Irradiations

### 4.1. Proton Irradiations

Samples 1 and 2 were irradiated in the beam course 32 at the CYRIC facility of the Tohoku University [10]. This facility provided a 70 MeV proton beam to the beam course in the intensity of 100 nA to 1300 nA, with a beam spot size of 7 mm of full width at half maximum (FWHM). The beam was scanned over the samples to get a uniform irradiation over the sample area. The particle fluence for each group of samples irradiated was measured by evaluating the activation of an aluminum foil for that group of samples irradiated. This method typically measures the dose for each irradiation step to roughly 10%.

### 4.2. Neutron Irradiations

**Figure 1:**
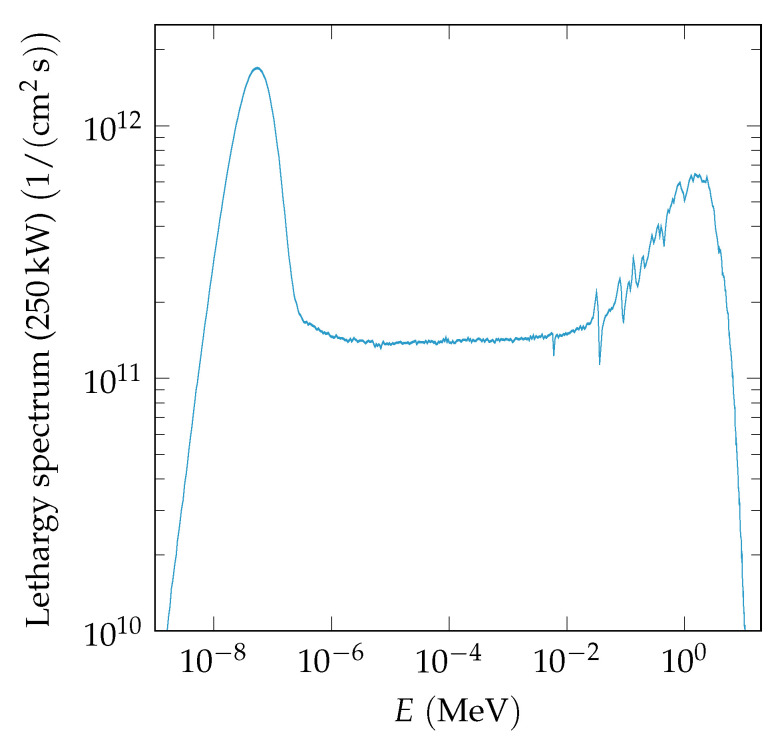
Lethargy neutron spectrum of channel F19 in Core 189 of the JSI TRIGA reactor used for all our neutron irradiations, at full reactor power (250 kW) [12].

Samples 3 and 4 were irradiated in channel F19 of Core 189 at the TRIGA nuclear reactor of the Jožef Stefan Institute (JSI) [11] with neutrons. At this facility, the neutron energy spectrum goes from 10^−8^ MeV to 7 MeV [11,12]. The neutron lethargy spectrum ($\log(E_{0}/E)$) of the F19 channel in Core 189, which was used for all irradiations, is shown in Figure 1. Fast neutrons are defined as having an energy greater than 0.1 MeV. The fast neutron spectrum peaks between 1 MeV and 2 MeV. Fast neutron fluxes up to 4 × 10^12^ *n*/(cm^2^ s) are available and were measured as a function of reactor power using gold foil activation [13]. To measure the fast neutron accumulated dose, the power of the reactor is set and the exposure time is recorded. This system typically measures the accumulated dose for each irradiation step to ∼10%.

### 4.3. Pion Irradiations

Samples 5, 6 and 7 were irradiated at PSI [14] with 200 MeV positive pions. Irradiations were performed by personnel from the CERN IRRAD facility [15]. The fluences were determined at CERN by measuring the activation in an aluminum foil mounted directly to each sample [16]. In addition to the statistical error, the CERN IRRAD website quotes an uncertainty of 20% for these measurements, due to the uncertainty on the hardness factor, which was added in quadrature to the statistical error.

## 5. Test Beam Analysis

The analysis of data in this work used the same analysis procedure and methods described in [4]. A brief description is given below and a detailed description can be found in [6,17].

**Figure 2:**
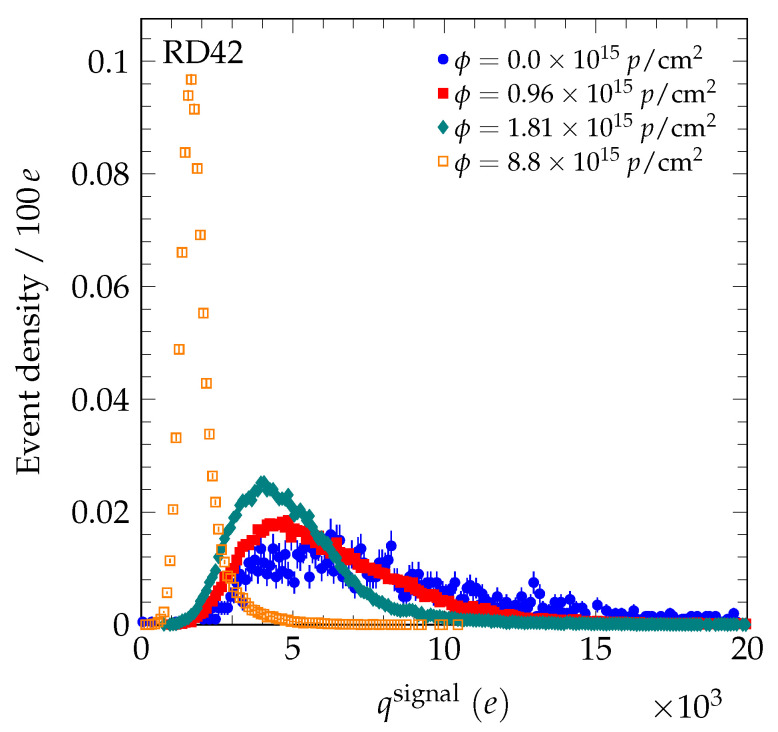
The signal charge spectrum evolution for samples irradiated with 70 MeV protons biased at E = +2 V/μm. The pulse height spectrum before irradiation was measured using a setup with a ^90^Sr β-source and a single pad metallization on the diamond. The integral of each spectrum has been normalized to unity.

**Figure 3:**
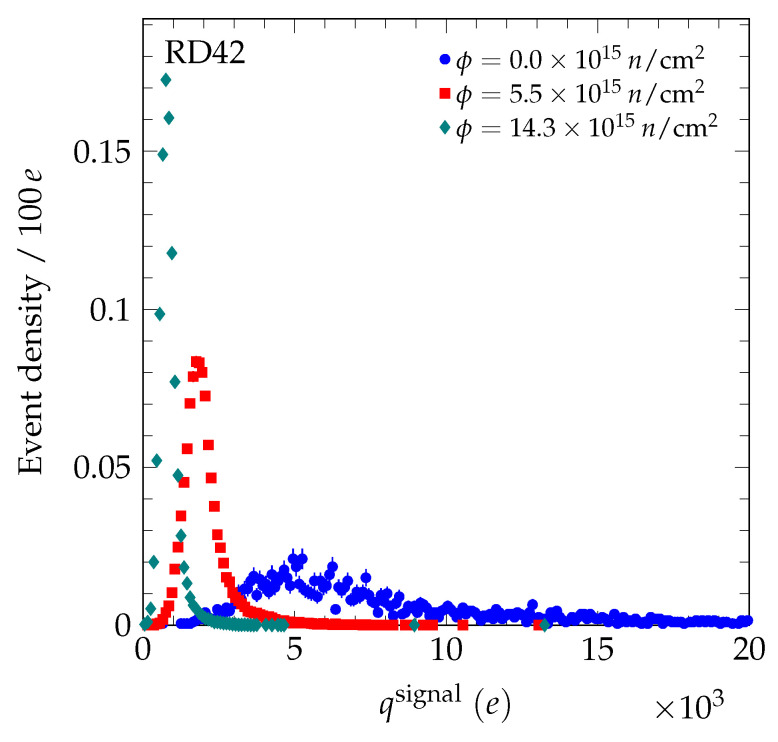
The signal charge spectrum evolution for samples irradiated with fast neutrons biased at E = −2 V/μm. The pulse height spectrum before irradiation was measured using a setup with a ^90^Sr β-source and a single pad metallization on the diamond. The integral of each spectrum was normalized to unity.

**Figure 4:**
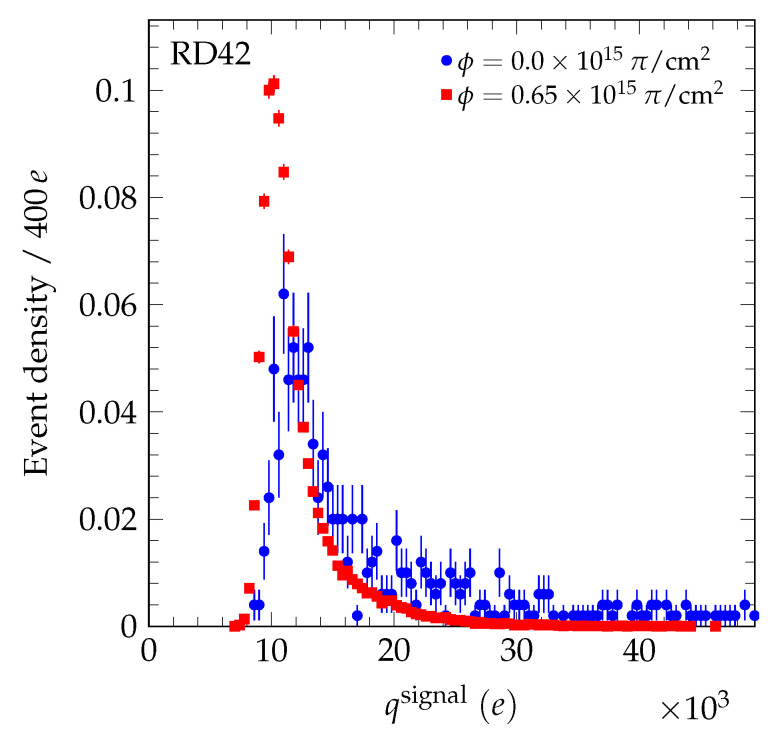
The signal charge spectrum evolution for the scCVD diamond sample irradiated with 200 MeV pions biased at E = +2 V/μm. The pulse height spectrum before irradiation was measured using a setup with a ^90^Sr β-source and a single pad metallization on the diamond, biased at 1 V/μm, since the detector collects all the charge at a bias voltage of 200 V. The integral of each spectrum was normalized to unity.

Data from an eight-plane silicon strip telescope [18], based on 50 μm pitch strip detectors with one or two floating intermediate strips, were used to reconstruct the predicted particle position in the diamond detector to roughly 1.3 μm. A transparent reconstruction algorithm was used to reconstruct the signal charge (and actual position) of the particle in the diamond [4]. In this algorithm, the charge on the highest five contiguous strips within a 500 μm window of the predicted particle position are summed to calculate the signal charge and actual position of the particle in the diamond detector. In Figure 2, we present the evolution of the measured signal charge spectrum after the 70 MeV proton irradiations. In Figure 3, the evolution of the measured signal charge spectrum after neutron irradiations is shown. In all cases, the signal charge spectra get narrower with fluence and the average value decreases with fluence. In Figure 4, the measured signal charge spectrum of an scCVD diamond sensor before and after pion irradiation is shown. The average value of the spectrum decreases with fluence. The same overall effects were observed previously in the 24 GeV and 800 MeV proton irradiations [4,19].

The average distance an electron–hole pair drifts apart under the influence of an applied electric field or “charge collection distance” (ccd) was calculated from the measured signal charge spectrum by

(1) $$\mathrm{ccd}=\bar{q^{\mathrm{signal}}}\times \frac{1\mu m}{36\;e}$$

where $\bar{q^{\mathrm{signal}}}$ is the average of the measured signal charge spectrum in units of *e* and 36 *e* is the average number of electron–hole pairs created per micron for a minimum ionizing particle (MIP). We measured this quantity by evaluating the signal response of an unirradiated scCVD diamond sample to a ^90^Sr β-source. After correcting the electronic gain, offset, and deposited charge, we determined the constant necessary to collect full charge. This measurement was performed for positive and negative bias polarity independently. The unirradiated scCVD diamond used was 497 μm thick. In a measurement cycle, we took data at multiple voltages up to ±500 V. In the source setup used, the electrons from ^90^Sr are 8% above minimum ionizing [9]. Our result, after all corrections, is (36.0±0.8) *e*/μm for positive bias voltage and (35.9±0.8) *e*/μm for negative bias voltage. These results are consistent with previous work [9,20,21]. The ccd can be expressed by [22]

(2) $$\frac{\mathrm{ccd}}{t}=\sum_{k=e,h}\frac{\lambda_{k}}{t}\left[1-\frac{\lambda_{k}}{t}\left(1-e^{-\frac{t}{\lambda_{k}}}\right)\right]$$

where *t* is the thickness of the diamond and $\lambda_{k}$ is the average distance an electron or hole drifts in an infinitely thick sample of a given material. Using Equation (2), the schubweg or average total distance the electron–hole pair moves apart, defined as the sum $\lambda =\lambda_{e}+\lambda_{h}$, was calculated for each beam test measurement from the measured ccd, assuming the ratio $\lambda_{h}/\lambda_{e}=1.3_{-0.6}^{+0.8}$ [4]. The effect of not knowing this ratio exactly was quantified in [4] and determined to not change the results of this analysis within the quoted errors.

## 6. Measurement of Damage Constants

After each irradiation step, the diamond devices were characterized in a 120 GeV hadron test beam at CERN. In a measurement cycle, the device under test was measured with both positive and negative bias voltage to obtain the sum of mean drift paths, λ, for an electric bias field of ±2 V/μm.

**Figure 5:**
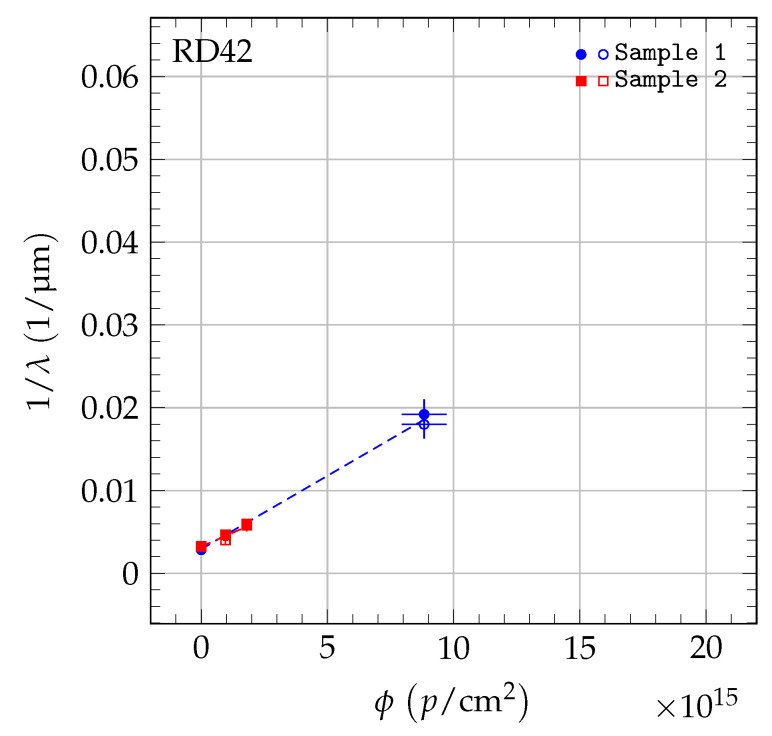
The 1/λ for pCVD diamond in the 70 MeV proton irradiation. The two values shown at each fluence are the values for positive (solid markers) and negative (open markers) bias at E= ±2 V/μm. The data were fit with a first-order damage curve independently for each sample.

**Figure 6:**
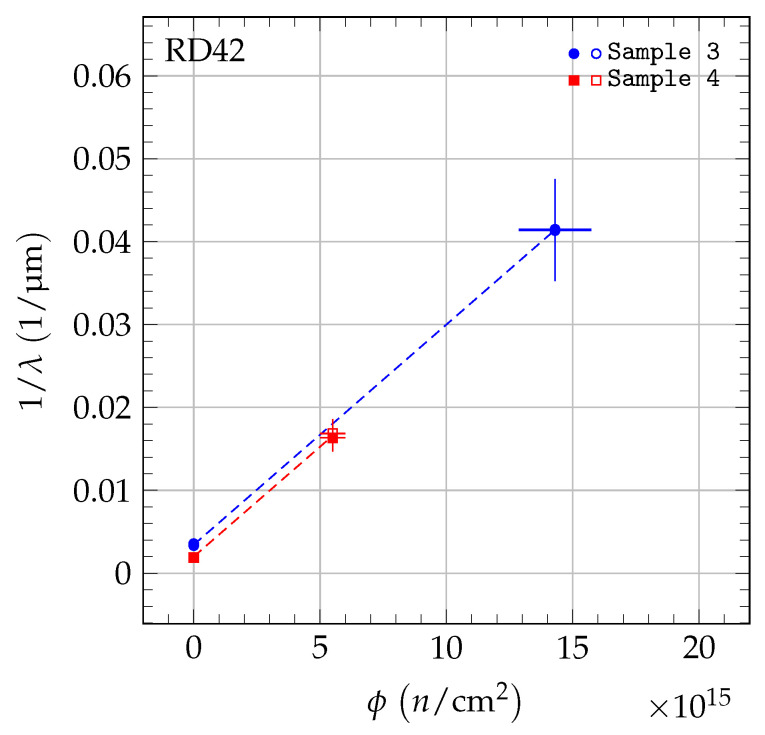
The 1/λ for pCVD diamond in the fast neutron irradiation. The two values shown at each fluence are the values for positive (solid markers) and negative (open markers) bias at E= ±2 V/μm. The data were fit with a first-order damage curve independently for each sample.

**Figure 7:**
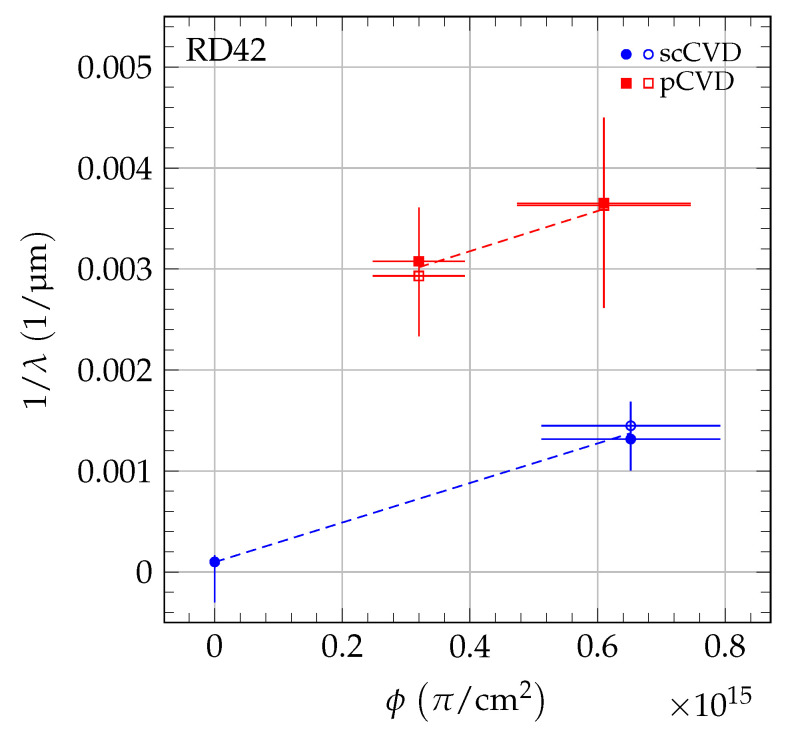
The 1/λ for scCVD and pCVD diamond in the pion irradiation. The two values shown at each fluence are the values for positive (solid markers) and negative (open markers) bias at E= ±2 V/μm. The data were fit with a simple damage curve independently for each diamond type. The uncertainty for unirradiated scCVD diamond comes from not knowing the upper initial mean drift distance exactly.

A first-order damage model was used to describe the irradiation damage effects. The model relates the inverse sum of mean drift paths, 1/λ, linearly with the irradiation fluence by [23]:

(3) $$\frac{1}{\lambda }=\frac{1}{\lambda_{0}}+k\phi$$

where *k* is the damage constant and $\lambda_{0}$ accounts for charge traps in the unirradiated state. The inverse sum of mean drift paths as a function of irradiation fluence is shown in Figure 5 for 70 MeV proton irradiations, in Figure 6 for fast neutron irradiations, and in Figure 7 for 200 MeV pion irradiations. For each sample, the damage model was fitted to the data points to derive the slope. The damage constant $k_{i}$ of particle species *i* was derived from the slopes of the individual samples. For the pion irradiated samples, the damage model was fitted separately to the data of scCVD and pCVD diamond samples irradiated with pions and the observed damage constants were combined. The final results of the damage constants for 70 MeV proton irradiations, fast neutron irradiations and 200 MeV pion irradiations are:

$$\begin{array}{cc} k_{\mathrm{pCVD}}^{\mathrm{proton}} & =1.62\pm 0.07\;\left(\mathrm{stat}\right)\pm 0.16\;\left(\mathrm{syst}\right)\times 10^{-18}\frac{{\mathrm{cm}}^{2}}{p\;\mu m}\\ k_{\mathrm{pCVD}}^{\mathrm{neutron}} & =2.65\pm 0.13\;\left(\mathrm{stat}\right)\pm 0.18\;\left(\mathrm{syst}\right)\times 10^{-18}\frac{{\mathrm{cm}}^{2}}{n\;\mu m}\\ k^{\mathrm{pion}} & =2.0\pm 0.2\;\left(\mathrm{stat}\right)\pm 0.5\;\left(\mathrm{syst}\right)\times 10^{-18}\frac{{\mathrm{cm}}^{2}}{\pi \;\mu m}. \end{array}$$

The general form of the statistical and systematic errors are given in [4]. Specifically, in this work, the statistical errors are dominated by the error in the fits while the systematic errors are dominated by the signal calibration, the pulse height dependence on track position and the pulse height dependence on bias polarity.

In Table 4, the relative damage constants are compared to the 24 GeV proton and 800 MeV proton results from [4] and to the 25 MeV proton results from [24]. The 70 MeV protons were found to be more than twice as damaging as 24 GeV protons; fast reactor neutrons were observed to be more than four times more damaging than 24 GeV protons; and 200 MeV pions were found to be more than three times as damaging as 24 GeV protons. These results are roughly consistent with displacement per atom (DPA) [25].

## 7. Universal Damage Curve

**Figure 8:**
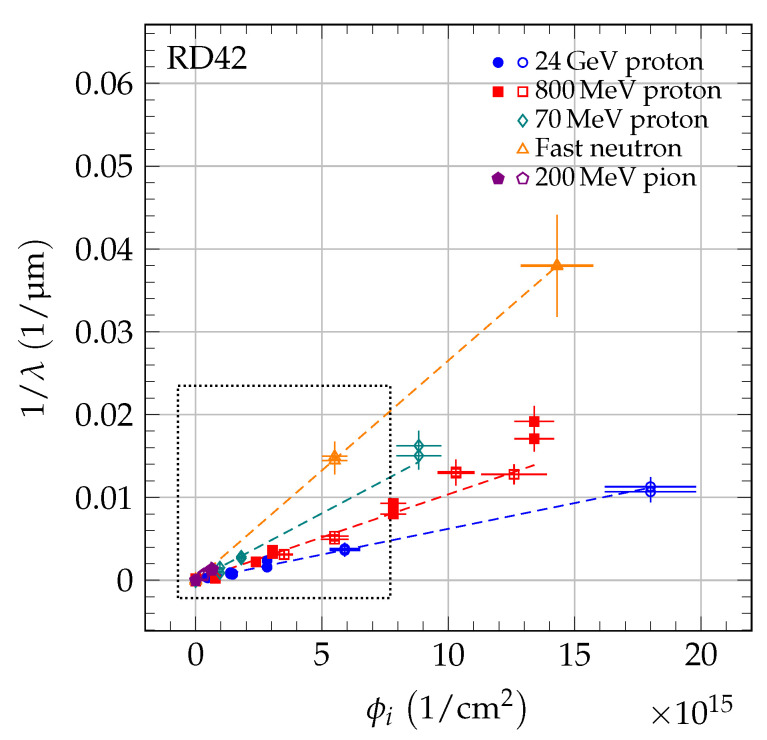
The 1/λ for scCVD (solid markers) and pCVD (open markers) diamond. As reference, the 800 MeV proton and 24 GeV proton data from [4] are plotted. Each point is shifted by $1/\lambda_{0,j}$. The dotted box indicates the zoom area shown in Figure 9.

**Figure 9:**
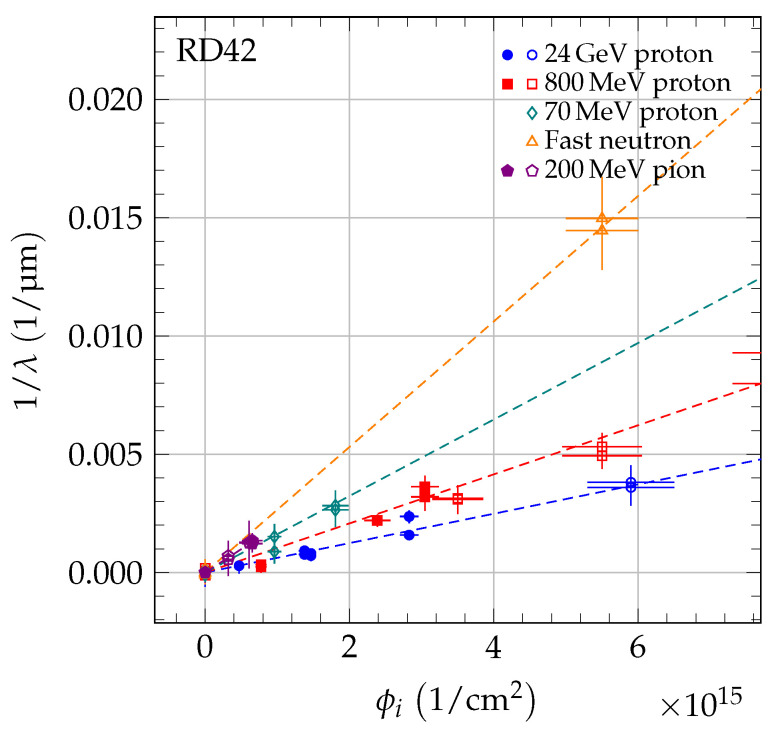
The 1/λ for scCVD (solid markers) and pCVD (open markers) diamond up to a fluence of 7 × 10^15^/cm^2^ (zoom of dotted box in Figure 8). As reference, the 800 MeV proton and 24 GeV proton data from [4] are plotted. Each point is shifted by $1/\lambda_{0,j}$.

As shown in [4], scCVD and pCVD diamond follow the same damage model. However, the damage curve of species *i* for each diamond sample *j* starts at a different value of $1/\lambda_{0,j}$, due to the initial collection distance of the sample. The initial $\lambda_{0,j}$ of sample *j* was derived by fitting a slope equal to the damage constant $k_{i}$ to the data points. Table 5 lists the parameter $\lambda_{0,j}$ of the tested samples. In Figure 8, the 1/λ as a function of fluence for 70 MeV proton, fast neutron, and 200 MeV pion irradiations is compared to the result of 24 GeV proton and 800 MeV proton irradiations [4] with the data points shifted by $1/\lambda_{0,j}$. The difference in slope of the dashed lines in this figure reflects the difference in damage constants. Figure 9 shows the data from Figure 8 in the dotted box to illustrate the relation of the low fluence data and the damage curves.

**Figure 10:**
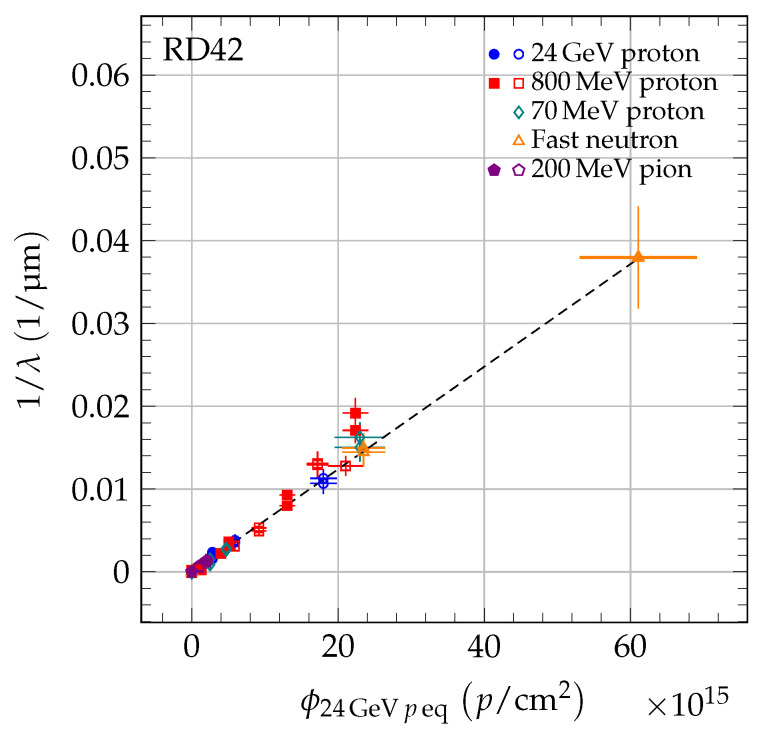
The 1/λ for scCVD (solid markers) and pCVD (open markers) diamond. Each point is shifted by $1/\lambda_{0,j}$. The fluence of each point was scaled by the relative damage constant, $\kappa_{i}$, to the 24 GeV proton equivalent fluence. The damage model (dashed line) is fitted to the data points.

Since scCVD and pCVD diamond follow the same damage model in Equation (3) and different irradiations have different shift, it should be possible to generate a universal damage curve. To accomplish this, the fluences were scaled by

(4) $$\phi_{24\;\mathrm{GeV}\;p\;\mathrm{eq}}=\kappa_{i}\times \phi_{i}$$

where $\kappa_{i}$ is the relative radiation damage constant defined as $\kappa_{i}=k_{i}/k_{24\;\mathrm{GeV}p}$. The measured 1/λ as a function of 24 GeV proton equivalent fluence is shown in Figure 10 and illustrates the universality of the first-order radiation damage model described above.

**Figure 11:**
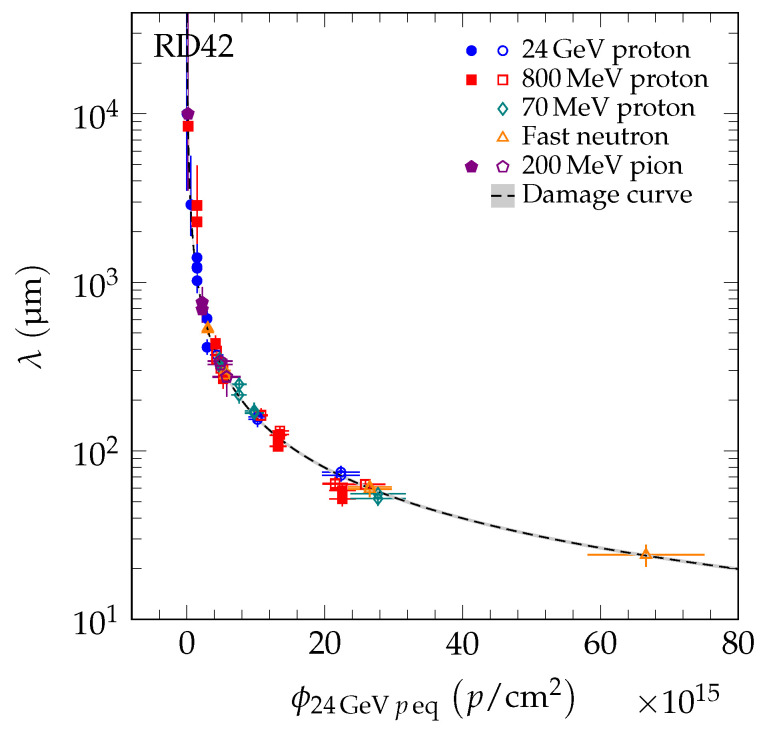
The λ for scCVD (solid markers) and pCVD (open markers) diamond. The fluence of each point was scaled by the relative damage constant to the 24 GeV proton equivalent fluence. Each point is shifted by $\phi_{0,j}$ which represents the starting value of sample *j* in 24 GeV proton equivalent fluence space. The dashed line is the fit of the damage model in Equation (3) to the data points. The gray band indicates the variation of the fit parameters by one standard deviation.

Instead of the offset $1/\lambda_{0,j}$, the separation of the damage curve of species *i* for individual diamond samples may be interpreted as a fluence offset, $\phi_{0,j}$. Thus, the initial signal response of pCVD diamond corresponds to irradiated scCVD diamond. The fluence offset in units of 24 GeV proton equivalent fluence of each diamond sample was calculated using:

(5) $$\phi_{0,j}=\frac{\kappa_{i}}{\lambda_{0,j}k_{i}}=\frac{1}{\lambda_{0,j}k_{24\;\mathrm{GeV}p}}$$

where $k_{i}$ is the damage constant for particle species *i* and $\lambda_{0,j}$ the initial sum of mean drift paths of sample *j* derived from the fit to the data points of sample *j* with fixed $k_{i}$. The fluence offset $\phi_{0,j}$ is listed in Table 5. To facilitate predictions of the signal response as a function of particle fluence, the measured λ of the five datasets were plotted as a function of 24 GeV proton equivalent fluence. To obtain a universal curve, the fluence of species *i* was scaled as in Equation (4) and each diamond data point was shifted by the fluence offset $\phi_{0,j}$. The measured λ as a function of 24 GeV proton equivalent fluence of the data is presented in Figure 11.

## 8. Measurement of the FWHM/MP Ratio

The initial non-uniformities in unirradiated pCVD material are mainly due to the interior crystal structure where single grains have different charge collection properties causing a spatial variation of the Landau-like distributions in the material [19]. This effect was clearly demonstrated in [4] where we showed the quantity *R*,

(6) $$R=\frac{\mathrm{FWHM}}{\mathrm{MP}},$$

is also a measure of the uniformity of the material. The smaller the quantity *R* the narrower the normalized signal charge distribution is across the material. Here, we used the full width at half maximum (FWHM) normalized by the most probable value (MP) of the signal response [4] to analyze its irradiation dependence (we normalized to MP, since the inherent distribution is Landau-like and for Landau distributions the measured mean depends on the number of events attained). To compare with previous beam test results for *R*, we used a +120GeV/c hadron beam, which is near minimum ionizing, as we did previously [4].

**Figure 12:**
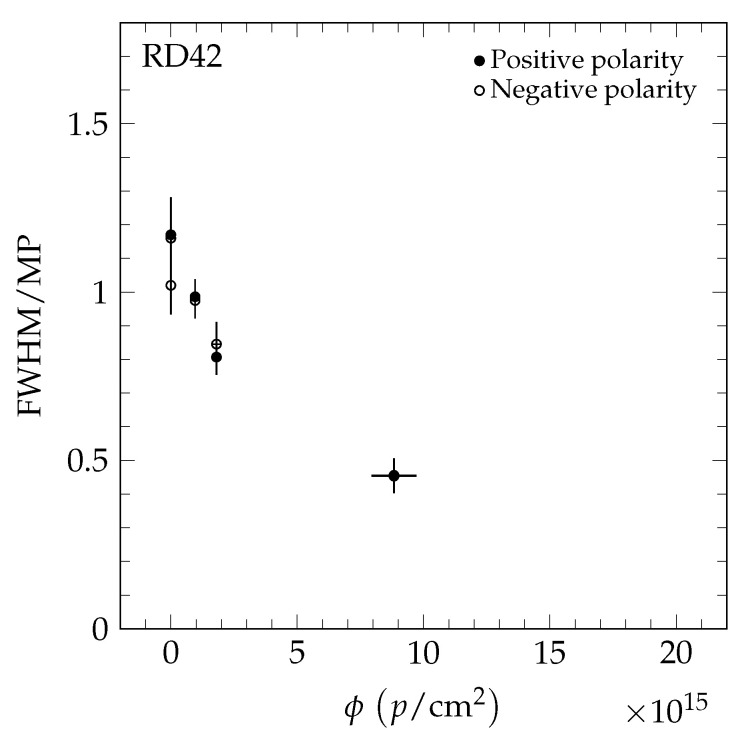
The FWHM/MP as a function of fluence in the 70 MeV proton irradiation measured in a +120 GeV/c hadron beam at CERN. The two values shown at each fluence are the values for positive (solid markers) and negative bias (open markers) at E=2 V/μm.

**Figure 13:**
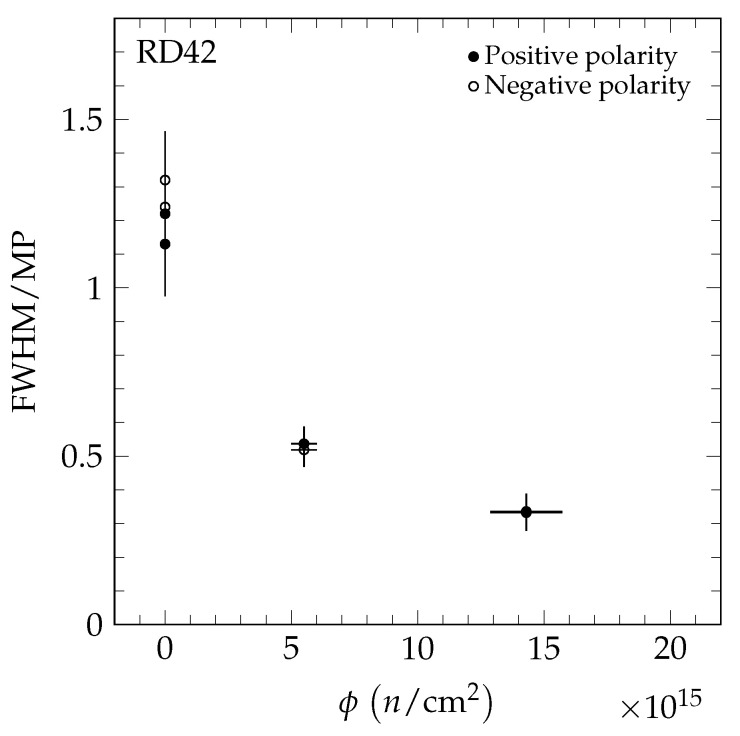
The FWHM/MP as a function of fluence in the fast neutron irradiation measured in a +120 GeV/c hadron beam at CERN. The two values shown at each fluence are the values for positive (solid markers) and negative bias (open markers) at E=2 V/μm.

**Figure 14:**
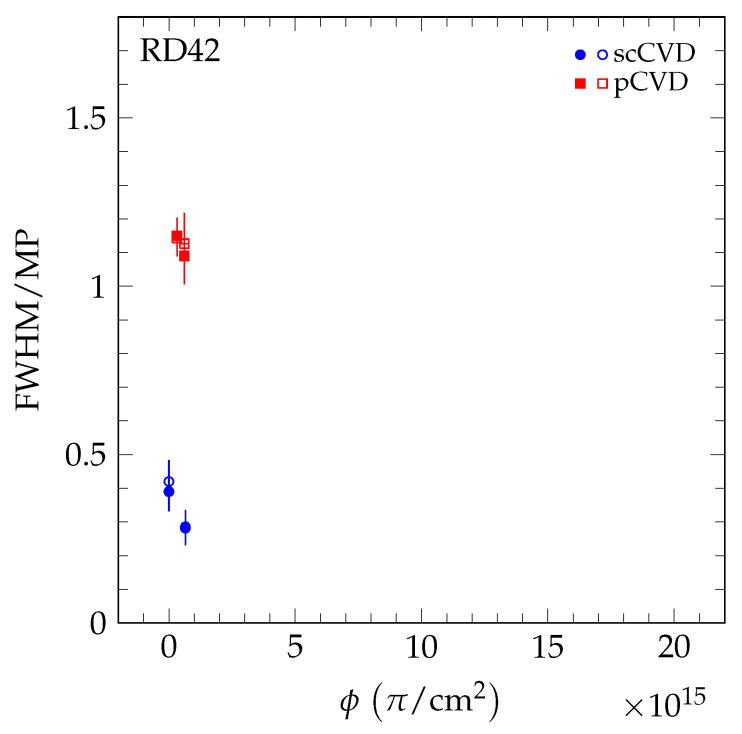
The FWHM/MP as a function of fluence in the pion irradiation measured in a +120 GeV/c hadron beam at CERN. The two values shown at each fluence are the values for positive (solid markers) and negative bias (open markers) at E=2 V/μm.

The results of the 70 MeV proton irradiations are shown in Figure 12. The results of the neutron irradiations are presented in Figure 13. The value *R* decreases for both irradiation species as a function of fluence, confirming the observation with pCVD diamond in [4]. The value *R* as a function of 200 MeV pion fluence is shown in Figure 14 and is compatible with the findings in [4].

**Figure 15:**
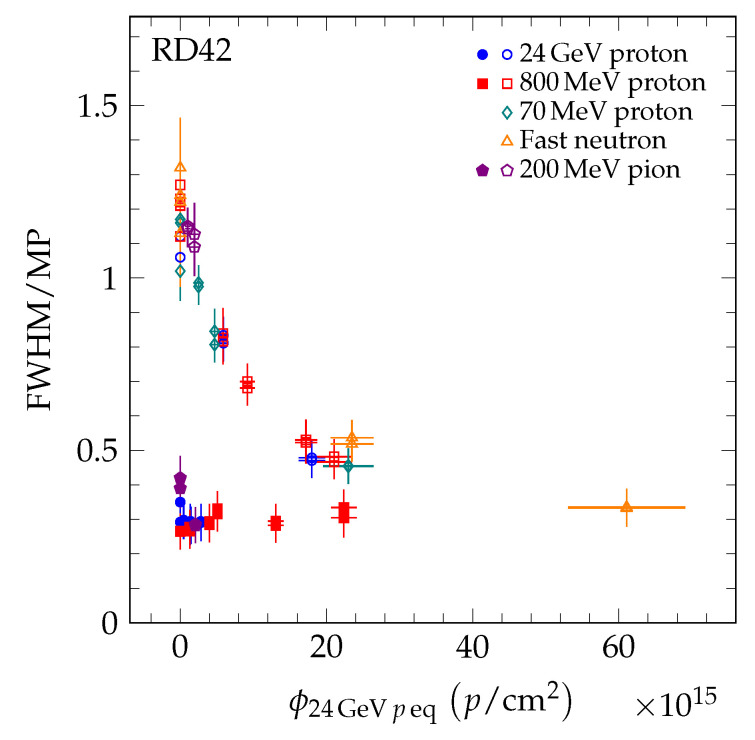
The FWHM/MP for scCVD (solid markers) and pCVD (open markers) diamond. The fluence of each point was scaled by the relative damage constant to the 24 GeV proton equivalent fluence.

**Figure 16:**
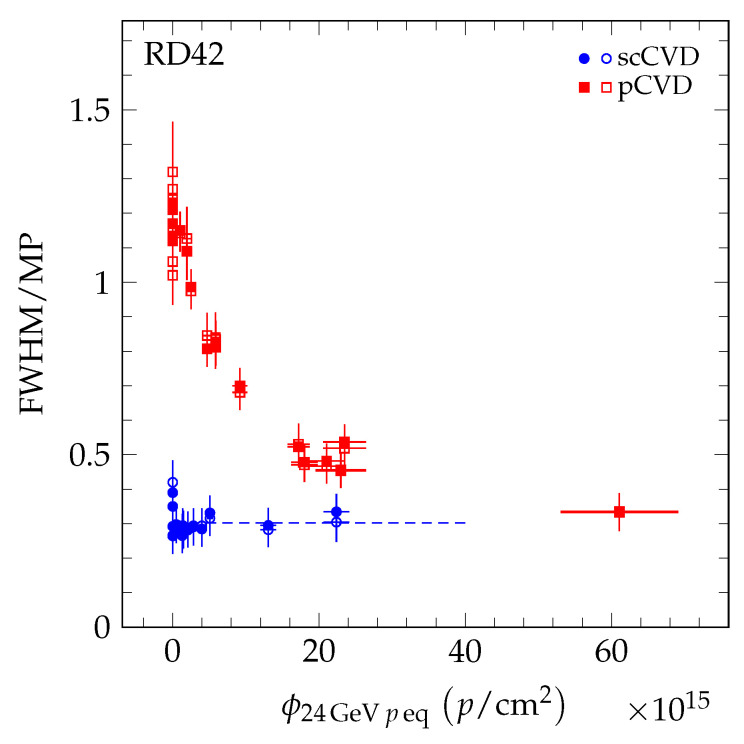
The FWHM/MP of scCVD and pCVD diamond samples irradiated with 24 GeV protons, 800 MeV protons, 70 MeV protons, fast reactor neutron, and 200 MeV pions for positive (solid markers) and negative bias (open markers) at E=2 V/μm. The fluence of each point was scaled by the relative damage constant to the 24 GeV proton equivalent fluence. The dashed line represents a constant fit to the scCVD diamond data points (blue) extrapolated to 40 × 10^15^ *p*/cm^2^ for illustrative purposes.

To compare the results of the different particle species, the fluence of each data point was scaled to 24 GeV proton equivalent fluence using Equation 4. In Figure 15, the value *R* of the five datasets (24 GeV protons [4], 800 MeV protons [4], 70 MeV protons, fast neutrons, and 200 MeV pions) is shown as a function of 24 GeV proton equivalent fluence. The pCVD data all fall on a single curve which decreases with fluence. The scCVD data all fall on a different curve, which is compatible with being flat with fluence. This is illustrated in Figure 16 where scCVD diamond data are shown in blue and pCVD diamond data are shown in red. The two curves taken together indicate the pCVD samples are becoming more uniform with irradiation and approaching the uniformity of single-crystal diamond.

## 9. Comparison with Silicon

Once the damage constants are determined, the damage constants for diamond may be compared with the damage constants for silicon. The collected charge in silicon devices depends on the electric field and trapping times. The trapping time $\tau_{i}$ at a temperature *T* and time after irradiation, *t*, is inversely proportional to the fluence [26,27]:

(7) $$\frac{1}{\tau_{i}}=\beta_{i}\left(T,t\right)\times \phi .$$

From the measurements of trapping times which require a fully depleted detector, the mean drift path of the charge carriers, λ, may be calculated using the relation

(8) $$\lambda =v_{e}\tau_{e}+v_{h}\tau_{h}$$

where $v_{i}$ are the drift velocities of electrons and holes, respectively. Using Equations (7) and (8), 1/λ as a function of fluence for silicon may be described by

(9) $$\frac{1}{\lambda }=\frac{1}{\frac{v_{e}}{\beta_{e}}+\frac{v_{h}}{\beta_{h}}}\times \phi =k\phi$$

where *k* is a damage constant.

**Figure 17:**
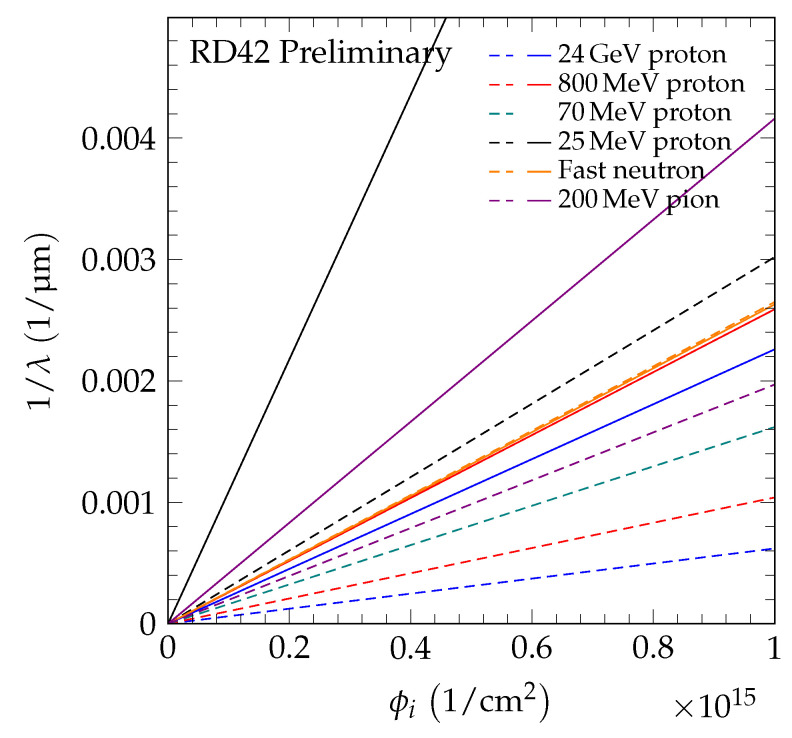
The 1/λ for CVD diamond and silicon for proton, neutron and pion irradiations at an electric field of 2 V/μm. The charge collection was measured at room temperature. The dashed lines are diamond results from this work and those in [4] for irradiations at 24 GeV protons (blue), 800 MeV protons (red), 70 MeV protons (green), fast neutrons (orange), and 200 MeV pions (purple), and the solid lines are the silicon damage data from RD50 [28] for proton (blue and red), neutron (orange), and pion (purple) irradiations. The curves for irradiations with 25 MeV protons were taken from [24].

As shown in Figure 17, the damage constants measured at an electric field of 2 V/μm and at $T=20\;{}^{{}^{\circ}}C$ for diamond and silicon were used to generate the inverse sum of mean drift paths versus fluence plot up to a fluence of 10^15^ particles/cm^2^. The dashed lines are the diamond results from this work and those in [4,24] for the irradiations of 24 GeV protons (blue), 800 MeV protons (red), 70 MeV protons (green), 25 MeV protons (black), fast neutrons (orange), and 200 MeV pions (purple). The solid lines are the silicon data from RD50 [28] for proton (blue and red), pion (purple) and neutron (orange) irradiations and data from [24] for 25 MeV proton (black) irradiations. The sum of mean drift paths is obtained from charge collection measurements at room temperature, assuming a uniform electric field of 2 V/μm. Drift velocities at 2 V/μm were derived from [29] and trapping times were taken from [27] after 80 min of annealing at 60 ${}^{{}^{\circ}}C$.

The results in Figure 17 show that for proton and pion irradiations diamond is much less radiation sensitive than silicon (greater than a factor of two) for all proton and pion energies measured, while for neutron irradiations silicon is comparable in radiation tolerance to diamond.

## 10. Summary

A study of CVD diamond material before and after a series of irradiations with 70 MeV protons, fast reactor neutrons and 200 MeV pions is presented. The decrease in signal response is in agreement with a first order damage model. The measured data were compared to previous measurements of CVD diamond samples irradiated with 800 MeV protons and 24 GeV protons [4]. Furthermore, the five datasets were combined in a universal damage curve for diamond material which allows predictions to be made for potential applications.

The decrease in FWHM/MP of the signal response of the collected charge as a function of particle fluence was confirmed for pCVD diamond material irradiated with 70 MeV protons and fast reactor neutrons. Moreover, the measurements presented in this paper were combined with previous measurements [4] into a universal curve.

Finally, the radiation damage constants of diamond were compared to the radiation damage constants of silicon. For proton irradiations, diamond was found to be more radiation tolerant than silicon, while a comparable radiation tolerance against neutrons was observed.

## Figures and Tables

**Table 1:** Properties of diamonds irradiated with 70 MeV protons and the fluence they received. The initial unirradiated Charge Collection Distance (ccd) values are given separately for positive and negative bias polarity (E=±2 V/μm).

Diamond	Type	Thickness (μm)	Area (mm${}^{2}$)	Initial *ccd* (μm)	Fluence (10${}^{15}$ *p*/cm${}^{2}$)
Sample 1	pCVD	518	10 × 10	227/238	0
					8.8 ± 0.9
Sample 2	pCVD	506	10 × 10	216/216	0
					0.96 ± 0.10
					1.81 ± 0.18

**Table 2:** Properties of diamonds irradiated with neutrons and the fluence they received. The initial unirradiated Charge Collection Distance (ccd) values are given separately for positive and negative bias polarity (E=±2 V/μm).

Diamond	Type	Thickness (μm)	Area (mm${}^{2}$)	Initial *ccd* (μm)	Fluence (10${}^{15}$ *n*/cm${}^{2}$)
Sample 3	pCVD	512	5 × 5	214/204	0
					14.3 ± 1.4
Sample 4	pCVD	510	10 × 10	295/292	0
					5.5 ± 0.5

**Table 3:** Properties of diamonds irradiated with pions and the fluence they received. The initial unirradiated Charge Collection Distance (ccd) values for the scCVD diamond sample are given separately for positive and negative bias polarity at 1 V/μm, since the detector collects all the charge at a bias voltage of 200 V. For Samples 6 and 7, only one bias polarity was measured for the unirradiated samples. For these samples, the initial ccd values are listed for comparison but were not used in the analysis.

Diamond	Type	Thickness (μm)	Area (mm${}^{2}$)	Initial *ccd* (μm)	Fluence (10${}^{15}$ π/cm${}^{2}$)
Sample 5	scCVD	497	5 × 5	497/497	0
					0.65 ± 0.14
Sample 6	pCVD	520	5 × 5	222	0
					0.32 ± 0.07
Sample 7	pCVD	508	5 × 5	228	0
					0.61 ± 0.14

**Table 4:** Relative damage constants for 24 GeV protons, 800 MeV protons, 70 MeV protons, 25 MeV protons, fast reactor neutrons, and 200 MeV pions. The radiation damage constants of 24 GeV protons and 800 MeV protons are from [4]. The relative radiation damage constant of 25 MeV protons is derived from [24].

Particle Species	κ
24 GeV protons	1.0
800 MeV protons	1.67 ± 0.09
70 MeV protons	2.60 ± 0.29
25 MeV protons	4.4 ± 1.2
Fast neutrons	4.3 ± 0.4
200 MeV pions	3.2 ± 0.8

**Table 5:** Parameter $\lambda_{0,j}$ and fluence offset $\phi_{0,j}$ of diamond sample *j* for 70 MeV protons, fast reactor neutrons, and 200 MeV pions. The derived $\lambda_{0,j}$ is not universal since it is sample dependent.

Diamond *j*	$\lambda_{0,j}$ (μm)	$\phi_{0,j}$ (10${}^{15}$/cm${}^{2}$)
Sample 1	340 ± 21	4.7 ± 0.6
Sample 2	318 ± 15	5.1 ± 0.6
Sample 3	291 ± 21	5.5 ± 0.7
Sample 4	531 ± 27	3.0 ± 0.4
Sample 5	11,000 ± 20,000	0.15 ± 0.31
Sample 6 + 7	420 ± 60	3.8 ± 0.7
